# Mucosa-associated lymphoid tissue lymphoma (MALT lymphoma) in the thyroid mimicking a painless subacute (De Quervain's) thyroiditis on presentation, fine needle aspiration and cytology, and ultrasound findings: A rare case report

**DOI:** 10.1016/j.ijscr.2023.108147

**Published:** 2023-04-11

**Authors:** Mohamed S. AL Hassan, Walid El Ansari, Adham Darweesh, Mouhammad Z. Sharaf Eldeen, Sarah Obiedat, Abdelrahman Abdelaal

**Affiliations:** a Department of General Surgery, Hamad General Hospital, Doha, Qatar; b Department of Surgery, Hamad General Hospital, Doha, Qatar; c College of Medicine, Qatar University, Doha, Qatar; d Weill Cornell Medicine–Qatar, Doha, Qatar; e Department of Clinical Imaging, Hamad General Hospital, Doha, Qatar; f Department of Laboratory Medicine & Pathology, Hamad Medical Corporation, Doha, Qatar

**Keywords:** Thyroid, Lymphoma, Mucosa-associated lymphoid tissue (MALT) lymphoma, Non-hodgkin, Primary thyroid lymphoma

## Abstract

**Introduction:**

We report a rare case of mucosa-associated lymphoid tissue lymphoma (MALT lymphoma) in the thyroid mimicking painless subacute (De Quervain's) thyroiditis.

**Presentation of case:**

Patient with history of hypothyroidism presented with huge non-tender goiter, compression symptoms and choking, no lymphadenopathy. Ultrasound (US) showed large thyroid lobes. There was a small hypoechoic nodule, and nonspecific lymphadenopathy. Fine needle aspiration/cytology (FNAC) of right thyroid nodule showed scant follicular cells, abundant polymorphic lympocytes, epithelioid histiocytes, and tingible body macrophages, suggestive of De Quervain's (granulomatous) thyroiditis. Total thyroidectomy was decided due to compression symptoms and huge goiter.

**Discussion:**

Intraoperative, thyroid was huge with no adhesions to the strap muscles/trachea. Total thyroidectomy with lymph node biopsy was undertaken. There were no complications. Postoperatively, the patient's condition was stable, breathing normally, and neck wound was clean. PTH was 11 pg/mL and calcium was 2.16 mmol/L, suggesting impending transient hypocalcemia. Histopathology showed lymphoepithelial lesions as clusters of lymphocytes within the thyroid follicles epithelium (MALT Balls). Immunohistochemical staining showed that the neoplastic lymphocytes were B cells and stained positive with B-cell markers CD20 and PAX5, but were negative for Cyclin D1 and for T cell markers CD3, CD5 and CD43. The patient was discussed at the lymphoma MDT meeting and the decision was to start the patient on radiotherapy which the patient received.

**Conclusion:**

Thyroid MALT lymphoma can mimic painless subacute thyroiditis. The triad of a large swelling of non-tender goiter with compression symptoms during a short period; FNAC findings suggestive of thyroiditis; and US showing enlarged thyroid lobes might cause confusion to the unsuspecting practitioner. Histopathology after excision provides definitive diagnosis.

## Highlights

• On occasions, a thyroid MALT lymphoma can mimic a painless subacute thyroiditis
• Triad of a large neck swelling of diffuse non-tender goiter with compression symptoms during a relatively short period; FNAC findings suggestive of thyroiditis; as well as US showing enlarged thyroid lobes might cause some confusion to the unsuspecting practitioner
• For such cases, MALT lymphoma needs to be in the differential diagnosis
• Histopathology after excision provides a definitive diagnosis. Treatment comprises surgery and radiotherapy where indicated, prognosis is generally good.

## 1 Introduction

Primary thyroid lymphomas (PTL) are rare thyroid neoplasms, characterized by a lymphomatous process involving the thyroid gland without spread or distant metastases from other areas of involvement at diagnosis [1]. PTL accounts for 0.5–5 % of all thyroid malignancies and 2.5–7 % of all extra nodal lymphomas [2], [3].

The most common types of PTL are mucosa-associated lymphoid tissue (MALT) lymphoma and diffuse large B-cell non-Hodgkin lymphoma (DLBCL). DLBCL accounts for >50 % of thyroid lymphomas, while MALT lymphoma is less common [4], [5], [6], [7]. A prerequisite for the occurrence of MALT lymphoma is the abundance of lymphoid tissue in the affected organ; therefore, it is usually seen in the salivary glands, the thyroid, eye, skin, and thymus gland [5].

The most common clinical symptom is painless progressively or sometimes rapidly enlarging neck swelling, and it might present with compressive features [1], [8], [9]. Although Fine needle aspiration and cytology (FNAC) evaluation of specimens is essential for diagnosis of PTL, findings might be confused with Hashimoto's thyroiditis [1]. PTL is virtually always diagnosed by a histopathological examination after surgical excision of the lesion [10]. Thyroid ultrasound and FNAC, using flow cytometry, remain the main modalities used to confirm the presence of lymphoma [11]. PTL necessitates accurate and early diagnosis, as its management is very different from that of other neoplasms intrinsic to the thyroid, and histopathological evaluation supplemented by immunohistochemistry is the gold standard for the diagnosis of PTL [12].

Thyroid MALT lymphoma is characterized by an indolent course, as most patients included in case reports and case series were alive with no evidence of disease [5]. Early stage intrathyroidal MALT lymphomas may be treated with surgery, radiotherapy, or a combination of both [11]. The prognosis is generally excellent but can vary because of the heterogeneous nature of thyroid lymphomas [11].

We report a 49-year-old male from Kenya diagnosed with thyroid MALT. The patient had a history of hypothyroidism and presented with a hugely diffuse non-tender goiter with compression symptoms and choking, but no lymphadenopathy. We report this case in line with the updated consensus-based surgical case report (SCARE) guidelines [13].

## 2 Case presentation

A 49-year-old male patient from Kenya presented at the thyroid surgery outpatient clinic of our institution (Hamad General Hospital, largest tertiary facility in Doha, Qatar), in July 2021. He presented with a painless neck swelling associated with compression symptoms in the form of difficult swallowing and choking since about 3 months. He had a history of hypothyroidism and was on levothyroxine. Upon physical examination, there was a hugely diffuse non-tender goiter, with no lymphadenopathy. The vitals were normal, and systems examination was unremarkable. There were no palpitations, no shortness of breath, cough, or wheezing. Family history was unremarkable. He was a current smoker.

**Fig. 1:**
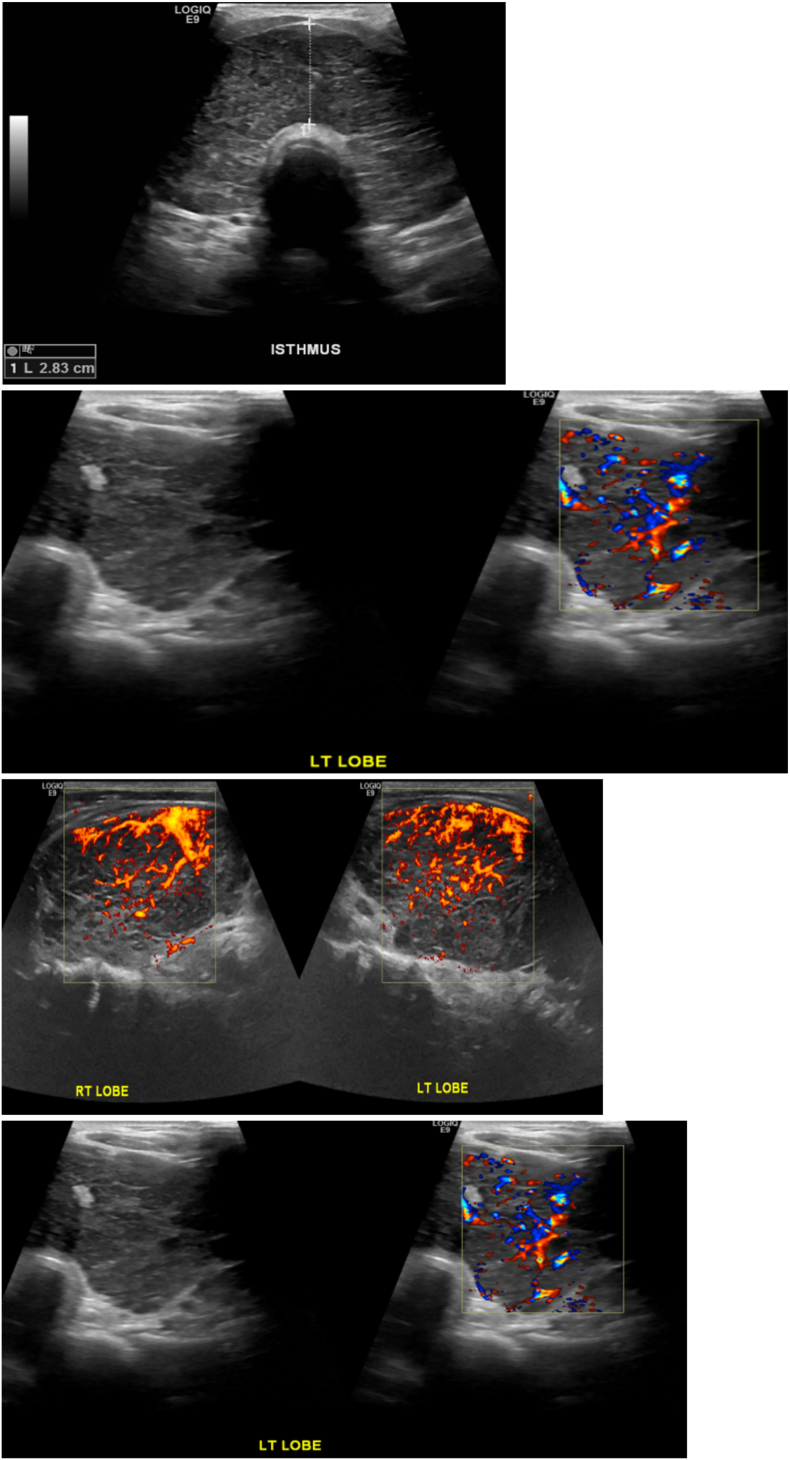
Thyroid ultrasound: thyroid parenchyma shows diffuse heterogenous echotexture pattern and increased vascularity.

**Fig. 2:**
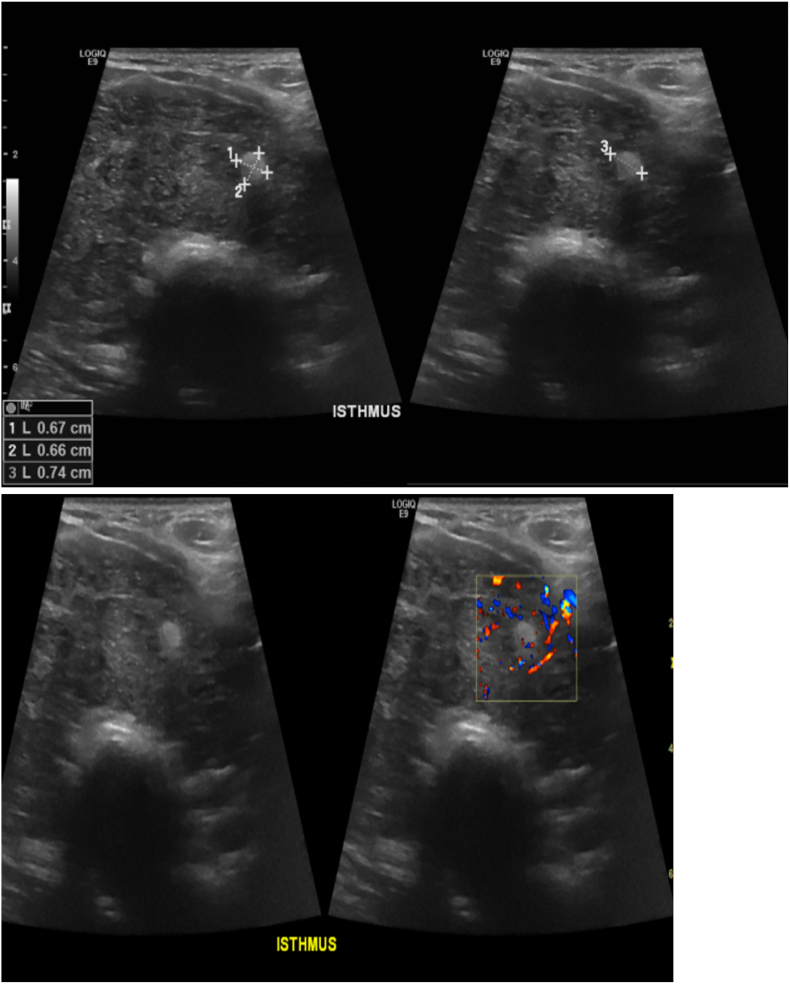
Thyroid ultrasound showing hyperechoic nodule measuring 0.74 cm in maximum dimension.

**Fig. 3:**
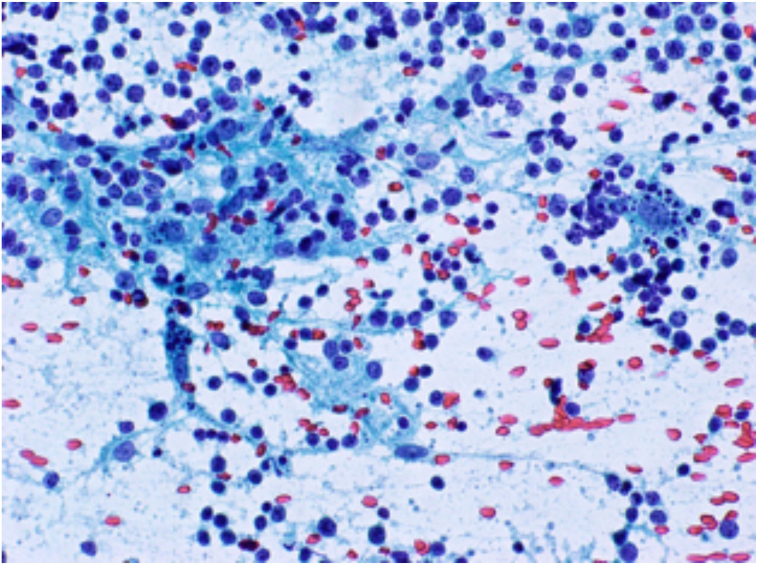
FNA cytology showing follicular cells, polymorphic lymphocytes, epithelioid histiocytes, tingible body macrophages and colloid.

Ultrasound (US) of the thyroid showed large right and left thyroid lobes (43.6 and 44.1 mm in maximal antero-posterior dimension respectively). There was a hypoechoic nodule (8 × 7 × 8 mm), and lymph nodes were noted, the largest at the right upper cervical region measuring 8.4 × 6.4 mm. Another lymph node was seen superior to the isthmus measuring 17 × 9 mm. These features suggested thyroiditis (Fig. 1, Fig. 2). FNAC of the right thyroid nodule showed scant follicular cells, abundant polymorphic lymphocytes, epithelioid histiocytes, tingible body macrophages and some colloid, suggestive of De Quervain's (granulomatous) thyroiditis (Fig. 3). The patient was started on conservative treatment for 2 months. The patient improved but the compression symptoms recurred, and total thyroidectomy was scheduled due to the gland's size, disfigurement of the neck, and the compression symptoms. This was discussed with the patient who agreed to the plan, and it was scheduled.

## 3 Surgical technique and intraoperative findings

**Fig. 4:**
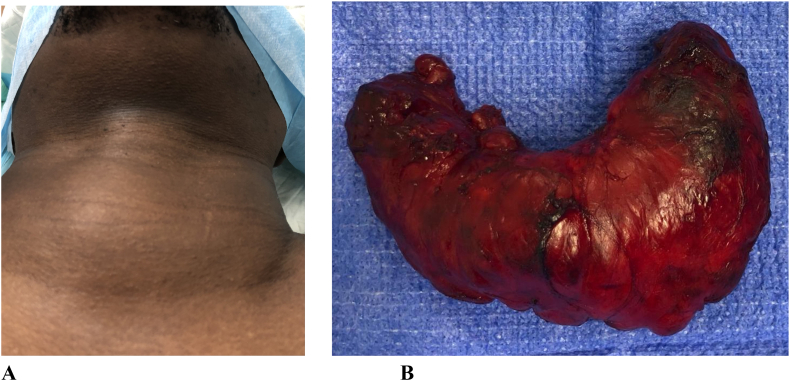
Huge thyroid before surgery (A), and the excised specimen (B).

Total thyroidectomy with central pre-tracheal lymph node biopsy was performed by a senior endocrine surgeon with the patient in supine position under general anesthesia (Fig. 4A). Transverse anterior neck incision with skin creases was undertaken. The right thyroid lobe was identified and dissected. Superior pole vessels were ligated individually, and cut, and the external branch of superior laryngeal nerve preserved, right recurrent laryngeal nerve identified and preserved. The same was done in the left lobe. Hemostasis was achieved, no drain was left, and wound was closed in layers.

Intraoperatively, the gland was huge with normal vascularity, with no adhesions to the strap muscles and trachea. There were no complications. Post operatively, the patient's condition was stable, breathing normally, and neck wound was clean with no swelling. However, PTH was 11 pg/mL and calcium was 2.16 mmol/L, suggesting impending transient hypocalcemia. The patient was discharged on day 2 and prescribed calcitriol, 0.5 μg oral capsules daily, calcium carbonate 1250 mg oral TID, and levothyroxine 100 μg oral tablets daily. The specimen (Fig. 4B) was sent for histopathology examination.

Total thyroidectomy with sentinel lymph node biopsy was done. The thyroid weighed 207 g, consisting of the right lobe (5 × 4 × 3 cm), left lobe (9 × 5 × 4 cm), and isthmus (5.5 × 4.5 × 2 cm). Slicing the specimen from superior to inferior revealed tan/pink hemorrhagic heterogenous cut surfaces with no discrete masses or nodules.

**Fig. 5:**
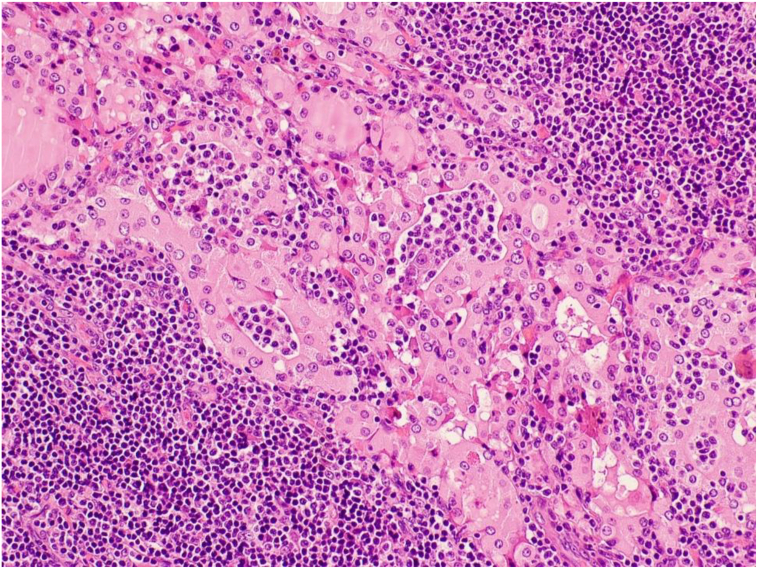
Medium power H&E photograph showing oncocytic changes in distorted thyroid follicles, infiltrated by clusters of lymphocytes forming lymphoepithelial lesions.

**Fig. 6:**
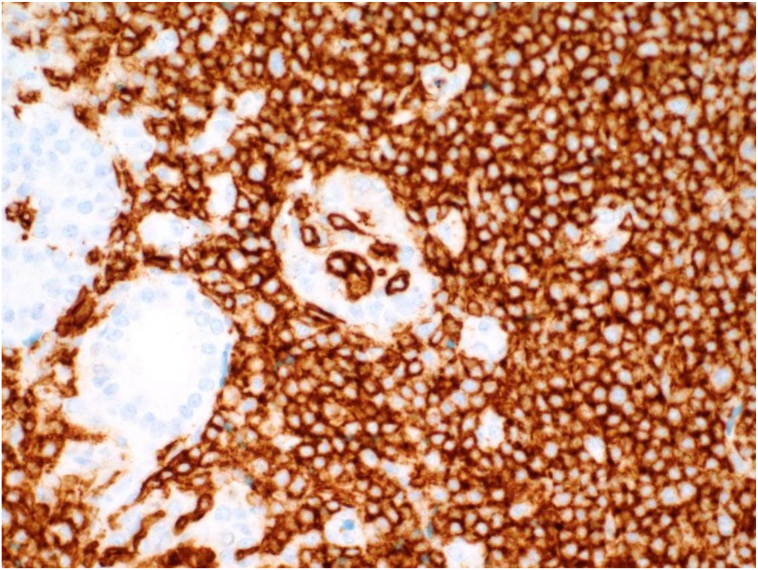
CD20 immunohistochemical stain is positive in the neoplastic B lymphocytes around the thyroid follicles and in the intraepithelial B lymphocytes.

**Fig. 7:**
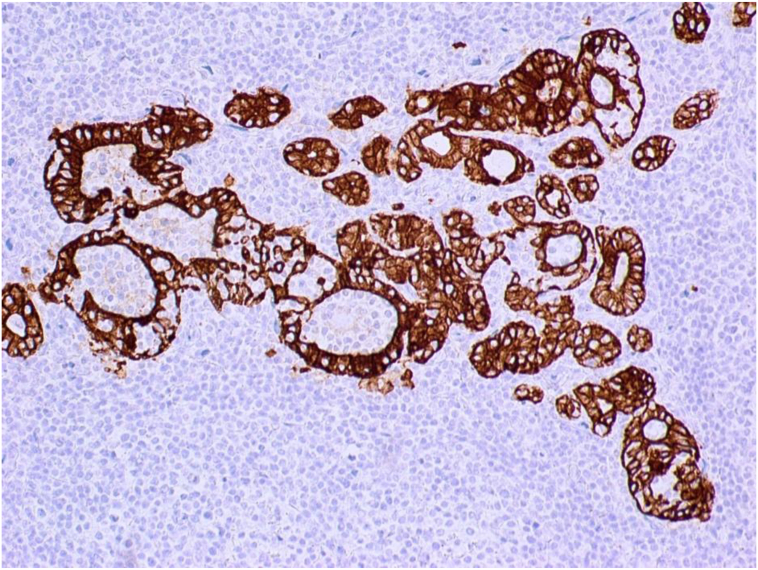
Cytokeratin immunohistochemical stain highlights the distorted thyroid follicle (positively stained) by the neoplastic lymphocytes (negatively stained).

Histologically (Fig. 5, Fig. 6, Fig. 7), sections of the thyroid showed architectural effacement by an atypical lymphoid infiltrate with a nodular pattern separated by thick fibrous bands. The infiltrate was composed of small centrocyte-like lymphoid cells with condensed nuclear chromatin, inconspicuous nucleoli and moderate amount of cytoplasm. Numerous plasmacytoid lymphocytes were present. Lymphoepithelial lesions, defined as clusters of lymphocytes within the thyroid follicles epithelium (MALT Balls), were easily identified. The background residual thyroid follicles showed variable squamous metaplasia and oncocytic changes.

By immunohistochemical stains, the neoplastic lymphocytes were B cells, stained positive with B-cell markers CD20 and PAX5. CD21 and CD23 showed expanded and distorted follicular dendritic cells meshwork. Cytokeratin AE1/AE3 confirmed the disrupted thyroid follicles and highlights the lymphoepithelial lesions. The proliferation marker Ki-67 labeling was low in the neoplastic lymphocytes.

The neoplastic lymphocytes were negative for Cyclin D1, and also negative for T cell markers CD3, CD5 and CD43. CD138 highlighted scattered plasma cells with polytypic staining for Kappa and Lambda.

Molecular PCR testing for IGH/TCR gene rearrangement showed no clear evidence of T or B cell clonal disease.

Although molecular testing for IGH/TCR gene rearrangement showed polyclonal B cells, however, the histologic and immunophenotypic findings were compatible with Mucosa-Associated Lymphoid Tissue Lymphoma (MALT-lymphoma).

Based on the histopathology reports, the patient was discussed at the thyroid MDT meeting, and the decision was to refer the patient to the lymphoma MDT meeting. At the lymphoma MDT meeting, the patient was classified as MALT lymphoma thyroid stage 1E, and the decision was to refer the patient to radiotherapy. The patient received 30 Gy in 15 fractions to the neck that was completed in August 2022 with no complications.

In terms of follow up, to date, the patient's recovery has been uneventful, he was symptom free and satisfied with the outcome of the surgery, calcium levels were normal, and he was on levothyroxine on and is being monitored without further treatment.

## 4 Discussion

MALT lymphoma is viewed as indolent and low grade PTL subtype [14], [15], [16]. We present a MALT lymphoma of the thyroid that mimicked painless subacute thyroiditis on presentation, FNAC and ultrasound.

As for demography, our case was a female in support of the literature, although a case series reported that PTL was of equal gender distribution [4], [17], [18], [19]. Our patient was 49 years old. The literature suggests an age of 63.7 (range: 51–74) years [4], [17]. However, patients with thyroid MALT lymphoma were younger than those with other types of thyroid PTL [19], concurring with the age of our patient (49 years old).

In terms of presentation, our case presented with a large neck swelling, supporting that the most frequent complaint in PTL is a large palpable neck swelling [4,18,]. The large mass caused our patient compression symptoms and choking, in agreement that a large PTL neck mass caused dysphagia, dyspnea, and hoarseness, which is typically associated with the disease [18].

As regards diagnosis, our patient's presentation, imaging and FNAC findings illustrate an interesting constellation. First, there was a history of neck swelling, compression symptoms and the case had a hugely diffuse non-tender goiter, with non-specific lymphadenopathy. These initial findings prompted a provisional thinking of subacute thyroiditis despite the fact that there was no pain, as subacute thyroiditis may rarely present as a solitary, painless nodule [20]. Secondly, our FNAC of the right thyroid nodule, showed scant follicular cells, abundant polymorphic lympocytes, epithelioid histiocytes, tingible body macrophages and some colloid, suggestive of De Quervain's (granulomatous) thyroiditis, a point that supported our initial provisional diagnosis. Such observation supports that while FNAC represents the gold standard to identify differentiated thyroid carcinoma, its reliability for the detection of PTL is still unclear. A meta-analysis by Zhang et al. demonstrated that FNAC has low sensitivity in diagnosing PTL, supporting the use of additional imaging and/or core biopsy when PTL is suspected [21]. Thirdly, US of our patient's thyroid showed that the right and left thyroid lobes were quite enlarged (maximal AP dimension 43.6 and 44.1 mm respectively). However, this massive enlargement could not be explained by the only small hypoechoic nodule (8 × 7 × 8 mm) identified by the US, further reinforcing the diagnoses of thyroiditis.

Hence, collectively these three features pointed to thyroiditis. Accordingly, our case was started on conservative treatment for 2 months. The patient improved but the compression symptoms recurred, and total thyroidectomy was scheduled due to the gland's size, disfigurement of the neck and the compression symptoms. However, post-op histopathologic examination showed numerous plasmacytoid lymphocytes as well as lymphoepithelial lesions, defined as clusters of lymphocytes within the thyroid follicles epithelium (MALT Balls). Only then was our diagnosis of thyroid MALT established. Our highlighted observations support others, where among four patients with PTL, two were diagnosed with fine needle aspiration biopsy (FNAB) and the other two were diagnosed with surgical excision [4]. Therefore, accurate diagnosis and rapid targeted treatment are essential for PTL cases [18].

As for management, although various treatments, e.g., surgery, chemotherapy, and radiotherapy have been employed alone or in combination for PTL, no clear guidelines seem to exist [19]. Surgery comprises the foundation management of rapidly growing thyroid tumors [19]. However, currently, its role is restricted to the immediate relief of the compression on the trachea and adjacent structures and is an essential element of palliative surgical management to ensure a patent respiratory tract before irradiation therapy [19]. Kikuchi et al. stated that Stage IE MALT lymphomas can be treated with radiotherapy or surgery alone; those with greater than stage IE MALT lymphoma should be treated with multidisciplinary therapy because they have a potentially poor outcome [22]. Overview on the management by Defrancesco and Arcaini reported that radiotherapy of the involved site is the favored choice for localized MALT lymphoma, as surgery alone cannot be considered if the resection margins are positive [23]. In agreement, our patient had a total thyroidectomy followed by radiotherapy to the neck with excellent outcome.

In terms of follow up, the patient's recovery has been uneventful. He is now symptom free, with normal calcium levels.

## 5 Conclusion

On occasions, thyroid MALT lymphoma can mimic a painless subacute thyroiditis. The triad of a large neck swelling of diffuse non-tender goiter with compression symptoms during a relatively short period; FNAC findings suggestive of thyroiditis; as well as US showing enlarged thyroid lobes might cause some confusion to the unsuspecting practitioner. For such cases, MALT lymphoma needs to be in the differential diagnosis as a possible condition. Histopathology after excision provides definitive diagnosis. Treatment comprises surgery first, followed by radiotherapy where indicated, and the prognosis is good.

## Consent

Written informed consent was obtained from the patient for publication of this case report and accompanying images. This is available for the Editor-in-Chief of this journal on request.

## Provenance and peer review

Not commissioned, externally peer-reviewed.

## Funding

Nothing to declare.

## Ethical approval

Approved by Medical Research Center, Hamad Medical Corporation reference number (MRC-04-22-499).

## Author contribution

Mohamed S. Al Hassan: study concept, data interpretation, editing and reviewing the paper. Walid El Ansari: study concept, data interpretation, writing the paper. Adham Darweesh: data interpretation, reviewing the paper. Sharaf Eldeen: histopathology interpretation, reviewing the paper. Sarah Obiedat: histopathology interpretation, reviewing the paper. Abdelrahman Abdelaal: study concept, data interpretation, writing and reviewing the paper. All authors read and approved the final version.

## Guarantor

Prof Dr Walid El Ansari.

## Research registration number

Not first in Man.

## Declaration of competing interest

Nothing to declare.

## References

[1] Agarwaf N., Wangnoo S.K., Sidiqqi A., Gupt M. (2013). Primary thyroid lymphoma: a series of two cases and review of literature. J. Assoc. Physicians India.

[2] Kossev P., Livolsi V. (1999). Lymphoid lesions of the thyroid: review in light of the revised european-american lymphoma classification and upcoming World Health Organization classification. Thyroid.

[3] Green L.D., Mack L., Pasieka J.L. (2006). Anaplastic thyroid cancer and primary thyroid lymphoma: a review of these rare thyroid malignancies. J. Surg. Oncol..

[4] Acar N., Acar T., Avcı A., Haciyanlı M. (2019). Approach to primary thyroid lymphoma: case series. Turk. J. Surg..

[5] Karvounis E., Kappas I., Angelousi A., Makris G.M., Kassi E. (2020). Mucosa-associated lymphoid tissue lymphoma of the thyroid gland: a systematic review of the literature. Eur. Thyroid. J..

[6] Kuper-Hommel M.J., Snijder S., Jansen-Heijnen M.L., Vreugdenhil A. (2005). Treatment and survival of patients with thyroid lymphoma: a population-based study with clinical and pathologic reviews. Clin. Lymphoma Myeloma.

[7] Stein S.A., Wartofsky L. (2013). Primary thyroid lymphoma: a clinical review. J. Clin. Endocrinol. Metab..

[8] Xie Y., Liu W., Liu Y., Wang W., Wang M. (2017). Diagnosis and clinical analysis of primary thyroid lymphoma. Zhongguo Yi Xue Ke Xue Yuan Xue Bao.

[9] Liu X.M., Ma D.L., Yuan G., Xie J.H. (2020). Progressively enlarging goiter: case reports of primary thyroid lymphoma and literature review. Curr. Med. Sci..

[10] Shah B.C., Ravichand C.S., Juluri S., Agarwal A., Pramesh C.S., Mistry R.C. (2007). Ectopic thyroid cancer. Ann. Thorac. Cardiovasc. Surg..

[11] Walsh S., Lowery A.J., Evoy D., McDermott E.W., Prichard R.S. (2013). Thyroid lymphoma: recent advances in diagnosis and optimal management strategies. Oncologist.

[12] Kakkar A., Purkait S., Agarwal S., Mallick S., Gogia A. (2019). Primary thyroid lymphoma: a series from a tertiary care center in northern India. J. Cancer Res. Ther..

[13] Agha R.A., Franchi T., Sohrabi C., Mathew G., Kerwan A. (2020). SCARE group, the SCARE guidelines: updating consensus surgical case report. Int. J. Surg..

[14] Chai Y.J., Hong J.H., Koo D.H. (2015). Clinicopathological characteristics and treatment outcomes of 38 cases of primary thyroid lymphoma: a multicenter study. Ann. Surg. Treat. Res..

[15] Ruggiero F.P., Frauenhoffer E., Stack B.C. (2005). Thyroid lymphoma: a single institution’s experience. Otolaryngol. Head Neck Surg..

[16] Derringer G., Thompson L., Frommelt A. (2000). Malignant lymphoma of the thyroid gland a clinicopathologic study of 108 cases. Am. J. Surg. Pathol..

[17] Uchida N., Yoshida M. (2020). Mucosa-associated lymphoid tissue (MALT) lymphoma developing in ectopic mediastinal thyroid tissue: a case report. Surg. Case Rep..

[18] Bostancı H., Dikmen K., Akyürek N., Büyükkasap A.Ç., Yavuz A., Yalçın M.M., Akın M. (2017). Eleven patients with primary thyroid lymphoma: a single center experience. Turk. J. Med. Sci..

[19] Chen E., Wu Q., Jin Y., Jin W., Cai Y., Wang Q. (2018). Clinicopathological characteristics and prognostic factors for primary thyroid lymphoma: report on 28 chinese patients and results of a population-based study. Cancer Manag. Res..

[20] Bianda T., Schmid C. (1998). De Quervain's subacute thyroiditis presenting as a painless solitary thyroid nodule. Postgrad. Med. J..

[21] Zhang L., Castellana M., Virili C., Crescenzi A., Giorgino F., Zucca E.E. (2019). Fine-needle aspiration to diagnose primary thyroid lymphomas: a systematic review and meta-analysis. Eur. J. Endocrinol..

[22] Kikuchi M., Shinohara S., Fujiwara K., Yamazaki H., Kanazawa Y. (2011). Eet al, clinical evaluation of 24 cases of primary thyroid malignant lymphoma. Nihon Jibiinkoka Gakkai Kaiho.

[23] Defrancesco I., Arcaini L. (2018). Overview on the management of non-gastric MALT lymphomas. Best Pract Res Clin Haematol..

